# Epidermolysis Bullosa Pruriginosa treated with baricitinib: A case report

**DOI:** 10.1097/MD.0000000000038854

**Published:** 2024-07-05

**Authors:** Zhe He, Qian Dong, Yue Xi, Rui Zheng

**Affiliations:** a Shanxi Medical University, Taiyuan, Shanxi, China; b Department of Dermatology, First Hospital of Shanxi Medical University, Taiyuan, Shanxi, China.

**Keywords:** baricitinib, Epidermolysis Bullosa, kinase inhibitors, treatment

## Abstract

**Introduction:**

Epidermolysis Bullosa Pruriginosa (EBP) is a persistent, recurring disease that seriously affects quality of life. Fewer than 100 cases of EBP have been reported to date. Numerous inflammatory dermatoses are driven by soluble inflammatory mediators, which rely on Janus kinase-signal transducer and activator of transcription (JAK-STAT) signaling, and inhibition of this pathway using Janus kinase (JAK) inhibitors might be a useful therapeutic strategy for these diseases.

**Patient concerns:**

A male patient, 28 years of age, was admitted to our hospital because of recurrent papules, nodules, and intense itching on the trunk and extremities for 12 years. Repeated large and intense itching has seriously affected the patient normal life.

**Diagnosis:**

The patient was diagnosed with EBP based on examination results.

**interventions:**

Oral baricitinib tablets (2 mg, once a day) + Oral desloratadine citrate disodium tablets (8.8 mg, once a day) combined with topical compound flumethasone ointment and Fucidin cream.

**outcomes:**

The patient skin rashes had subsided and flattened remarkable, and his itching was markedly relieved. The visual analogue scale (VAS) itching score of the patient gradually declined from 8 to 9 points to 2 to 3 points.

**Conclusion:**

This study confirms that baricitinib is effective and feasible in treating EBP, especially in remarkable relieving itching, which rendered new ideas for therapeutic approaches for EBP in the future.

## 1. Introduction

Epidermolysis Bullosa Pruriginosa (EBP), named in 1994 after the report by McGrath, is a distinct clinical subtype of Dystrophic Epidermolysis Bullosa (DEB), is characterized by nodular prurigo-like lichenoid lesions with intense itching, in addition to the features of DEB, such as blisters and onychodystrophy.^[1]^ In most cases, skin rashes are distributed over lower extremities, forearms, elbows, dorsum of hands, shoulders, and lower back, especially the extensor aspect of the extremities. Currently, EBP is treated with topical corticosteroids, tacrolimus, and oral thalidomide, but the outcomes are often unsatisfactory.^[2]^ Baricitinib is a competitive inhibitor of the Janus Kinase (JAK) family of non-receptor protein kinases, predominantly acting against JAK-1 and JAK-2 subtypes.^[3]^ Approved for severe cases of alopecia areata and moderate-severe atopic dermatitis in adults. baricitinib is being increasingly tried across many other indications with promising results. It has results suggest that JAK1 or JAK1/2 inhibitors could be a promising treatment option for DEB-related pruritus. Long-term safety should be assessed in future studies. Here, 1 case of baricitinib in the treatment of EBP is discussed.

## 2. Case presentation

**Figure 1.**
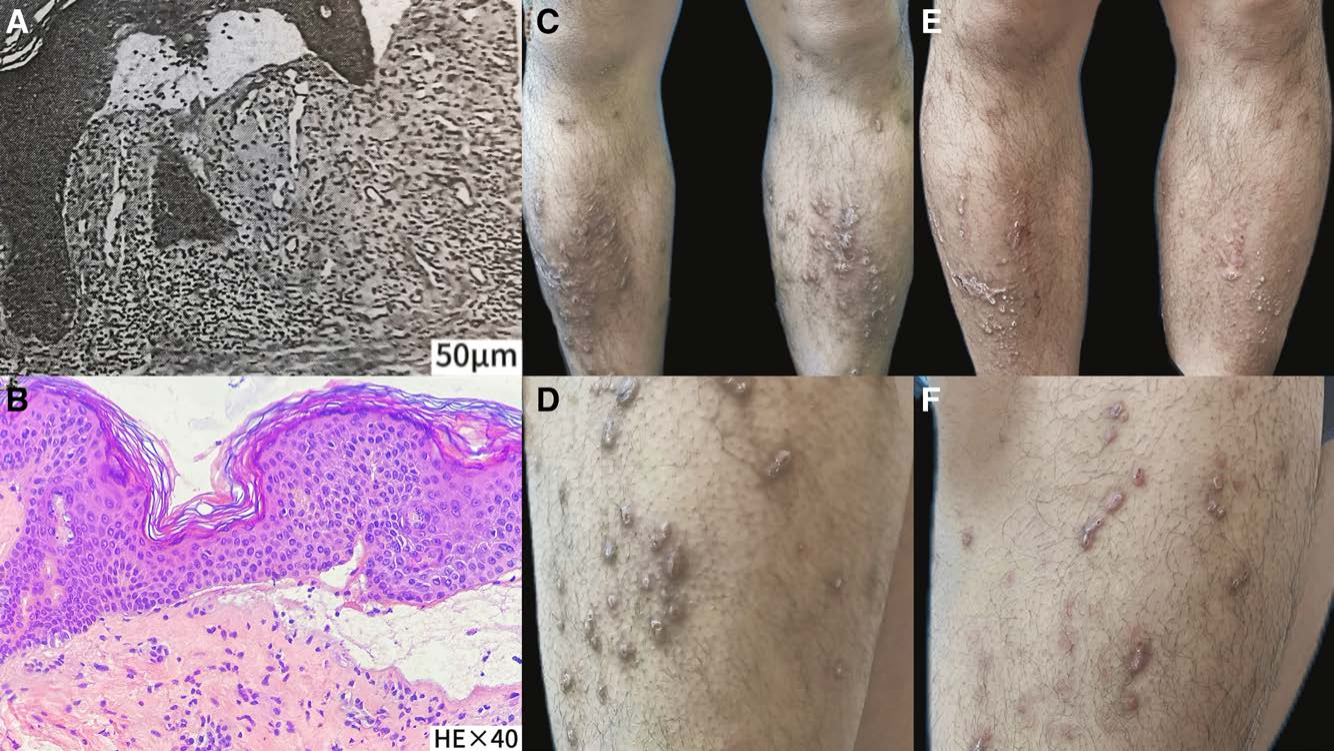
Hyperkeratosis, epidermal hyperplasia, and fissures can be seen at the junction of the true epidermis, inflammatory cells, mainly lymphocytes, were seen around the vessels in the superficial dermis (A, scale bar: 50 μm). Epidermis showed subepidermal bulla. Dermis shows mixed inflammatory infiltrate (B, HE × 40). multiple reddish-brown hemispherical mung bean-to-soybean-sized nodules were symmetrically distributed on the bilateral calves and right thigh, densely packed in patches, some of which were anabrotic and crustosus due to the intense itching (C and D). After 2 yr of baricitinib treatment, the results uncovered that the number of skin rashes on the outer side of both thighs had decreased by half, and the color had become dull, although some dark brown hyperpigmentation and scars remained on the extensor aspect of both lower legs (E and F).

A male patient, 28 years of age, was admitted to our hospital because of recurrent papules, nodules, and intense itching on the trunk and extremities for 12 years. The patient had visited another hospital 12 years ago (in 2010). He was diagnosed with EBP based on examination results from other hospitals (Fig. 1A) and herpetic autoantibody test results.

**Figure 2.**
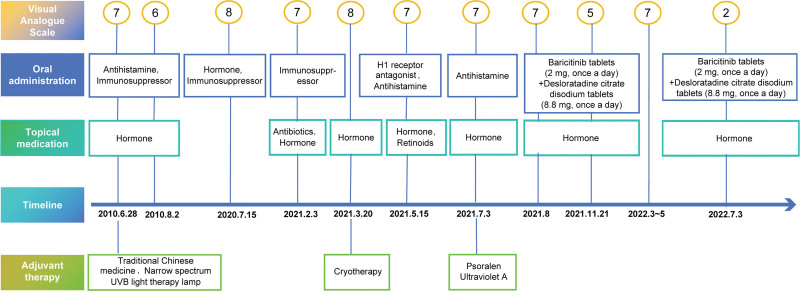
The patient prior medication timeline, as well as documented VAS scores after treatment with baricitinib, showed a gradual decline. VAS = visual analogue scale.

The patient had visited other hospitals several times due to disease relapse, but the outcomes were often unsatisfactory. The patient came to our hospital on August 2, 2021. The physical examinations revealed multiple reddish-brown hemispherical mung bean-to-soybean-sized nodules were symmetrically distributed on the trunk and extremities, densely packed in patches, some of which were anabrotic and crustosus due to scratching (Fig. 1C and D). Laboratory test results indicated routine blood and urine tests with normal liver and kidney function, blood function, blood electrolytes, and blood sedimentation were normal. We did a pathology biopsy on him again (Fig. 1B). He underwent symptomatic treatment with oral baricitinib tablets (2 mg, once a day) + oral desloratadine citrate disodium tablets (8.8 mg, once a day) combined with topical compound flumethasone ointment and Fucidin cream since August 2, 2021. The visual analogue scale (VAS) scale for the degree of itching was adopted to assess the severity and control of the disease. The VAS score of the patient was 7 to 8 points at the initial diagnosis. During the 2 years of treatment with baricitinib, the VAS itching score had steadily decreased (Fig. 2), and the patient reported significant improvement in itching and sleep quality after treatment, without any adverse effects. After discontinuation of baricitinib for 2 weeks, in July 2022, the patient returned to the clinic for reexamination, and the results indicated that the number of skin rashes on the outer side of both thighs had decreased by half, and the color had become dull, although some dark brown hyperpigmentation and scars remained on the extensor aspect of both lower legs (Fig. 1E and F). The results of regular reexaminations showed routine blood and urine with normal liver and kidney function and electrolytes. Currently, the patient is still under follow-up.

## 3. Discussion

DEB is an autosomal dominant or recessive genodermatosis caused by variations in COL7A1.^[4]^ Its pathogenesis is not completely understood. EBP is a distinct clinical subtype of DEB, characterized by nodular prurigo-like lichenoid lesions with intense itching.^[5]^ Currently, EBP is treated with topical corticosteroids, tacrolimus, and oral thalidomide, but the outcomes are often unsatisfactory. Baricitinib is a reversible selective inhibitor of tyrosine protein kinase, which is capable of modulating the signal transduction of helper T-cells (Th1, Th2, Th17, and Th22) and participates in many immune-mediated disorders.^[6]^

This case clinically presented with dense nodular, keratotic papules, mainly on the extensor side of both lower limbs, which were brownish in color, with umbilical concavity at the center of some of them, and subepidermal blisters could be seen by the results of the examination, while there were no obvious eosinophils in the blisters, and there was mixed inflammatory cell infiltration in the dermis, so we considered this to be a case of a specific type, EBP. Janus kinase-signal transducer and activator of transcription (JAK-STAT) is an intracellular signaling pathway upon which many different proinflammatory signaling pathways converge. Numerous inflammatory dermatoses are driven by soluble inflammatory mediators, which rely on JAK-STAT signaling, and inhibition of this pathway using JAK inhibitors might be a useful therapeutic strategy for these diseases. Evidence suggests that the dysregulation of helper T-cell signal transduction is implicated in epidermolysis bullosa. Chronic inflammation is a hallmark of DEB, thus upregulation of inflammatory cytokines and JAK signaling may play a role in DEB-related pruritus.

Caroppo reported 1 case of EBP who was successfully treated with dupilumab.^[7]^ Dupilumab can dually block and inhibit both IL-4 and IL-13 and suppress Th2-mediated inflammatory responses, thus remarkable improving skin lesions and relieving itching. Recent studies have indicated that type 2 inflammation plays a role in the pathophysiology of EBP.^[8]^ Wu XG et al reported 3 patients with EBP, Immunotyping of Th1/2/17 cell subsets in peripheral blood by flow cytometry revealed a higher Th2 but parallel Th1 and Th17 cell subsets in patients compared to healthy controls.^[9]^ This suggests that EBP may be triggered by Th2 immune mechanisms. Dysregulated Th2 cytokine signaling perpetuates inflammation and tissue damage. Baricitinib, a selective inhibitor of JAK1 and JAK2, exerts its therapeutic effects by interfering with the JAK-STAT signaling pathway, crucial for cytokine-mediated immune responses. Baricitinib suppresses the activation of JAK-mediated pathways downstream of interleukin-4 (IL-4), interleukin-13 (IL-13), and other pro-inflammatory cytokines implicated in the pathogenesis of EBP. By inhibiting Th2 cytokine-driven inflammation, baricitinib mitigates blister formation, pruritus, and disease progression in EBP.^[10]^ JAK may be a favored target for EBP-associated symptoms. Moreover, considering the affordability and durability of the treatment regimen for patients, baricitinib has become the preferred treatment choice in our clinic instead of dupilumab. Jiang XY et al reported 1 case of EBP in a 40-year-old man without a family history of DEB and with severe skin lesions and intense itching, which is remarkable improved after treatment with baricitinib.^[11]^ He was followed up once every 2 weeks until 16 weeks, and then every 8 weeks. The scores of all 3 indicators decreased over time. Joo Kwon retrospectively reviewed the medical records of DEB patients with refractory pruritus who were treated with either baricitinib, a JAK1/2 inhibitor, or upadacitinib, a selective JAK1 inhibitor. A total of 12 DEB patients (six recessive DEB and 6 dominant DEB) were included in this study. The mean ± SD baseline pruritus visual analog scale score was 7.5 ± 1.7. Upadacitinib or baricitinib treatment resulted in a rapid and sustained decrease in itch.^[12]^

In this paper, 1 case of EBP was discussed. The patient had a long history of EBP and a relevant family history of the disease. The typical skin lesions initially manifested as multiple lichenoid papules and nodules on both lower extremities, especially their extensor aspect, with scars forming in the center of the larger nodules, accompanied by mild scale. Combined with the fact that the results of previous examinations, the herpetic autoantibody test, and other examinations were all negative, prurigo nodularis and herpes could be excluded. The patient had intense itching, which is one of the important manifestations of EBP. The patient had received traditional treatment for more than 10 years, including oral antihistamines such as loratadine tablets, immunosuppressants such as thalidomide and topical fluticasone propionate cream and other hormonal drugs, in addition to trying to use Chinese medicine to treat, and while the progression of the disease had been controlled to a certain extent, the outcome of itching relief was unsatisfactory (Fig. 2). The patient was treated with baricitinib in our hospital with the hope of relieving the itching. During the 2 years of treatment with baricitinib, the patient skin rashes had subsided and flattened remarkable, and his itching was markedly relieved. The VAS itching score of the patient was assessed at follow-up, and the point plot showed that the score had gradually declined from 8 to 9 points to 2 to 3 points, indicating a greatly improved quality of life.

## 4. Conclusion

EBP is a persistent, recurring disease that seriously affects quality of life. this study confirms that baricitinib is effective and feasible in treating EBP, especially in remarkable relieving itching, which rendered new ideas for therapeutic approaches for EBP in the future. Particularly important for patients suffering from severe itchiness.

## Acknowledgments

I would like to thank Dr Rui Zheng for her kind guidance on this paper.

## Author contributions

**Conceptualization:** Zhe He.

**Data curation:** Zhe He.

**Formal analysis:** Rui¸ Zheng, Qian Dong, Yue Xi.

**Investigation:** Zhe He, Rui¸ Zheng.

**Methodology:** Rui¸ Zheng, Qian Dong, Yue Xi.

**Software:** Zhe He.

**Supervision:** Zhe He, Rui¸ Zheng, Qian Dong, Yue Xi.

**Writing – original draft:** Zhe He, Rui¸ Zheng.

**Writing – review & editing:** Rui¸ Zheng.
